# Population Pharmacokinetics Analysis of Amikacin Initial Dosing Regimen in Elderly Patients

**DOI:** 10.3390/antibiotics10020100

**Published:** 2021-01-20

**Authors:** Hideo Kato, Suzanne L. Parker, Jason A. Roberts, Mao Hagihara, Nobuhiro Asai, Yuka Yamagishi, David L. Paterson, Hiroshige Mikamo

**Affiliations:** 1 Department of Clinical Infectious Diseases, Aichi Medical University, Aichi 480-1195, Japan; katou.hideo.233@mail.aichi-med-u.ac.jp (H.K.); hagimao@aichi-med-u.ac.jp (M.H.); nobuhiro0204@gmail.com (N.A.); y.yamagishi@mac.com (Y.Y.); 2 University of Queensland Centre for Clinical Research, The University of Queensland, Royal Brisbane & Women’s Hospital, Brisbane, QLD 4029, Australia; suzanne.parker@uq.edu.au (S.L.P.); j.roberts2@uq.edu.au (J.A.R.); d.paterson1@uq.edu.au (D.L.P.)

**Keywords:** population pharmacokinetics analysis, amikacin, elderly, *Pseudomonas aeruginosa*

## Abstract

There are limited data of amikacin pharmacokinetics (PK) in the elderly population. Hence, we aimed to describe the population PK of amikacin in elderly patients (>70 years old) and to establish optimized initial dosing regimens. We simulated individual maximum concentrations in plasma (Cmax) and minimal concentrations (Cmin) for several dosing regimens (200–2000 mg every 24, 48, and 72 h) for patients with creatinine clearance (CCr) of 10–90 mL/min and analyzed efficacy (Cmax/minimal inhibitory concentration (MIC) ≥ 8) for MICs of 4, 8, and 16 mg/L and safety (Cmin < 4 mg/L). A one-compartment model best described the data. CCr was the only covariate associated with amikacin clearance. The population PK parameter estimates were 2.25 L/h for clearance and 18.0 L for volume of distribution. Dosing simulations recommended the dosing regimens (1800 mg) with dosing intervals ranging 48–72 h for patients with CCr of 40–90 mL/min based on achievement of both efficacy for the MIC of 8 mg/L and safety. None of the dosing regimens achieved the targets for an MIC of 16 mg/L. We recommend the initial dosing regimen using a nomogram based on CCr for an MIC of ≤8 mg/L in elderly patients with CCr of 40–90 mL/min.

## 1. Introduction

The proportion of the elderly (over 70 years old) is growing rapidly and has been predicted to exceed 20% of the worldwide population by 2050 [1], 1.93 times larger than the current population [2]. With an increasing proportion of the elderly come concerns for the requirements for optimal healthcare and treatment needs of this population group. Patients over the age of 70 years accounted for over 90% of patients hospitalized for pneumonia in one report [3]. Furthermore, two studies have reported that this population has a higher risk of acquiring nosocomial infections [4]. One of those studies found an increased association between acquiring infection with multidrug-resistant Pseudomonas aeruginosa and age > 70 years [5]. Aging results in various physiological changes to the human body and these changes can alter the pharmacokinetic properties of antibiotics. A reduction in renal function can impact on the clearance of some antimicrobials. A reduction in body water and an increase in fat content can alter the volume of distribution of some antimicrobials, both alterations necessitating altered dosing strategies [6].

Amikacin is an aminoglycoside antibiotic commonly used to treat serious, hospital-acquired infections. Amikacin exerts a rapid and potent bactericidal effect against Gram-positive and Gram-negative pathogens by concentration-dependent activity [7]. The effect has been strongly associated with the ratio of a maximum concentration (Cmax) to a minimum inhibitory concentration (MIC; Cmax/MIC) as the pharmacodynamic (PD) index related to a favorable clinical response [8]. A Cmax/MIC ratio of ≥8 has been established as a predictor of therapeutic success [9]. On the other hand, nephrotoxicity is the most common adverse event reported from amikacin use and is caused by the accumulation of amikacin in the proximal tubule cells [10]. The toxicity has been related to a high minimum concentration (Cmin) [7,11]. Some studies and the Japanese guideline have proposed a threshold of Cmin at 4 mg/L for an increased likelihood of nephrotoxicity [12,13,14].

In general, the optimal dosing regimen of amikacin is complicated by its narrow therapeutic index. Amikacin population pharmacokinetics (PK) studies have often been reported in pediatric [12,15,16,17,18,19,20,21,22] and adult [13,23,24,25,26] populations. Although one research was conducted in geriatric patients, the population consisted of patients aged 59–95 years [27]. Hence, the aim of this study is to describe the population PK of amikacin in elderly patients aged over 70 years, for the purpose of establishing maximally safe and effective initial dosing regimens through use of Monte Carlo simulations.

## 2. Results

### 2.1. Clinical Data

The demographics and clinical characteristics of the patients participating in this study are presented in Table 1. During the study period, 15 patients met the inclusion criteria. The mean age and creatinine clearance (CCr) were 80.6 y [range, 71–95 y] and 52.9 mL/min [range, 10.9–94.9 mL/min], respectively. The mean amikacin dose administered was 440 mg [range, 200–1000 mg].

The patients had pneumonia (*n* = 5), bacteremia (*n* = 5), urinary tract infection (*n* = 1), urinary tract infection and pneumonia (*n* = 1), pneumonia and bacteremia (*n* = 1), peritoneum inflammation and bacteremia (*n* = 1), and febrile neutropenia (*n* = 1). The samples studied microbiologically were positive for 11 patients (73.3%). The most common species isolated was Pseudomonas aeruginosa (*n* = 6: one metallo-β-lactamase-producing isolate; three isolates with carbapenem and quinolone resistance), followed by methicillin-resistant coagulase-negative staphylococci (*n* = 3: two Staphylococcus epidermidis; one Staphylococcus capitis), Klebsiella pneumoniae (*n* = 1: one metallo-β-lactamase-producing isolate), and Corynebacterium striatum (*n* = 1). The MIC of the Gram-positive isolates ranged from 0.25 to 32 mg/L, and that of the Gram-negative isolates ranged from 1.5 to 8 mg/L.

The 30-day mortality was 40.0% (6/15), and the microbiological eradication rate was 63.6% (7/11). Moreover, the achievement rate of Cmax/MIC of ≥ 8 for the survived patients was higher than that for dead patients (83.3% vs. 40.0%, *p* = 0.1368), and the rate for the patients who showed microbiological eradication was significantly higher than that for the patients who did not (85.7% vs. 25%, *p* = 0.0440).

### 2.2. Pharmacokinetic Modeling

**Figure 1:**
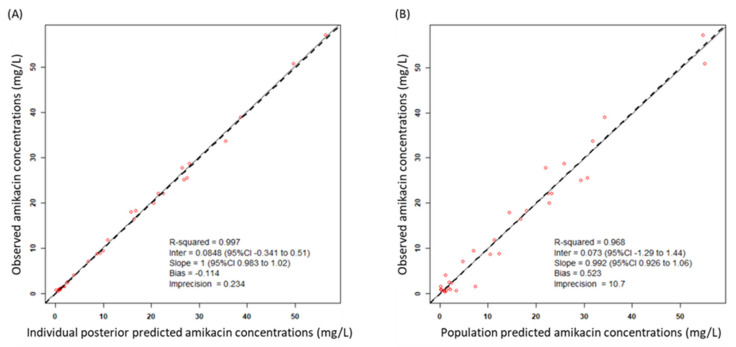

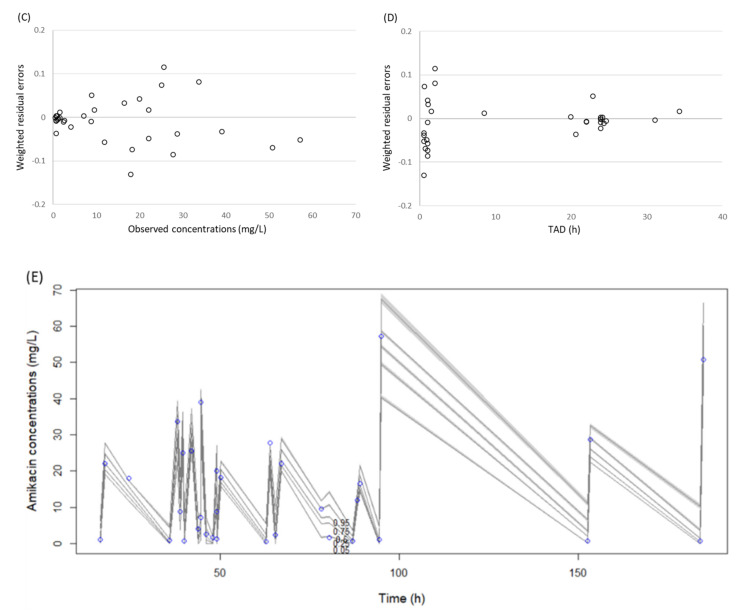
Diagnostic plots for the final covariate model. (**A**) Observed vs. individual posterior predicted amikacin concentrations and (**B**) observed vs. population predicted amikacin concentrations in plasma. The solid lines represent the line of best fit (regression line). CI, confidence interval; Inter, intercept. (**C**) Weighted residuals errors vs. observation concentrations and (**D**) weighted residuals errors vs. time after dose (TAD). (**E**) Observed amikacin concentration time data and visual predictive check of the final model. The lines represent concentrations at the designated quantile given by the number on the line (0.0.5, 0.25, 0.5. 0.75, and 0.95). The circles represent observed patient amikacin concentrations.

A total of 33 amikacin concentrations were obtained from 15 patients, with 2 to 3 serum samples collected per patient. For all of the patients, two or three serum samples were collected. Samples were drawn at 30 min after the end of infusion (Cmax) and immediately before the administration of the next amikacin dosage (Cmin). We tested one- and two-compartment models and the results suggest that the one-compartment linear model described the data adequately and was most parsimonious. This model consisted of the primary PK parameters of clearance (CL) and volume of distribution in the central compartment (Vd). One covariate of CL was identified as improving the fit of the base model with CCr providing a correlation of r^2^ = 0.5. Inclusion of CCr in the model was normalized to the median value of the study population of 52.1 mL/min. After the inclusion of CCr in the base model, the −2 log-likelihood value decreased by 5.9. Moreover, the Akaike information criterion (AIC) value decreased and the goodness of fit improved (Figure 1A–D). Based on these conditions, CCr was accepted for inclusion in the final model as a covariate to CL.

The final model was described as follows: amikacin CL = CLs × (CCr/52.9), where CLs is clearance in elderly patients. The mean population PK parameter estimates from the final model were 2.25 ± 0.78 L/h for CL and 18.0 ± 3.4 L for Vd (Table 2). A visual predictive check plot based on *n* = 1000 simulations with the final model is shown in Figure 1E. The proportion of observations between the 5th and 95th simulated percentiles was 90.9% and this was deemed acceptable.

### 2.3. Probability of Target Attainment (PTA)

Using the Monte Carlo simulation, the PTA of efficacy (Cmax/MIC ≥ 8) against MICs of 4, 8, and 16 mg/L according to various dosing regimens was simulated, and the PTA of safety (Cmin < 4 mg/L) for various dosing regimens for patients with CCr of 10–90 mL/min was simulated (Table 3). All dosing regimens tested for patients with CCr of < 30 mL/min were not able to achieve both a Cmax/MIC ≥ 8 and a Cmin < 4 mg/L in more than 90% of patients for an MIC of 4 mg/L. As the best dosing regimens for an MIC of 4 mg/L, patients with CCr of 30–39, 40–79, and over 80 mL/min achieved the PK/PD targets in ≥90% at 800 mg q72h, 800 mg q48h, and 800 mg q24h, respectively. Moreover, all dosing regimens tested for patients with CCr of < 40 mL/min were not able to achieve both PK/PD targets in more than 90% of patients for an MIC of 8 mg/L. As the best dosing regimens for an MIC of 8 mg/L, patients with CCr of 40–59 and over 60 mL/min achieved the PK/PD targets in ≥90% at 1800 mg q72h and 1800 mg q48h, respectively. In contrast, none of the dosing regimens for patients with CCr of 10–90 mL/min showed the achievement of both the PK/PD targets in more than 90% of patients for MICs of 16 mg/L (Table 3).

### 2.4. Fractional Target Attainment

The fractional target attainment for simulated PTAs for various dosing regimens for patients with CCr of 10–90 mL/min for P. aeruginosa is shown in Table 4. All dosing regimens to achieve the priori PTAs of both Cmax/MIC of ≥ 8 and Cmin of < 4 mg/L for patients with CCr 10–90 mL/min achieved fractional target attainment at over 90% (range: 93.0–94.2%). Of these dosing regimens, patients with CCr of 40–59 and over 60 mL/min achieved the fractional target attainment in ≥ 85% at 1800 mg q72h and 1800 mg q48h, respectively.

## 3. Discussion

We have previously recommended the optimal dosing regimens of amikacin for the initial dosage for patients over the age of 15 years [13]. Moreover, multiple studies on an optimal dosing regimen of amikacin using population PK analysis have been reported in patients with a specific condition such as pediatric patients or patients with different renal functions on continuous renal replacement therapy [28,29]. However, there is no report on optimal dosing regimens using population PK analysis in elderly patients.

To date, the population PK of amikacin has been described by either a one-compartment [28,30] or a two-compartment model [13,25,29]. Data collected in our study demonstrate that there was no improvement in the log-likelihood value using the two-compartment model and the one-compartment model was accepted. Furthermore, the final model in our study shows that CCr has a statistically significant influence on amikacin CL. Several studies have reported the influence of CCr on the PK of amikacin, and this covariate has been incorporated into various population models for amikacin [13,23,25,29,30]. No covariate was associated with the volume of distribution of amikacin. In other studies, covariates identified as significantly associated with the volume of distribution were body weight-related factors [13,23,25,28,29,30] and hypoalbuminemia [31]. These covariates were not included in our study. Our population PK model showed PK parameter estimates of 0.05 L/h/kg and 0.40 L/kg for CL and Vd, respectively. A previous study evaluating the PK of amikacin among elderly patients reported an estimate of CL of 0.04 L/h/kg and an estimate of Vd of 0.33 L/kg [32]. In contrast, estimates of CL and Vd in young adult patients under the age of 70 years were 0.10–0.15 L/h/kg and 0.23 L/kg, respectively [33,34]. Amikacin is eliminated almost entirely through the kidneys by glomerular filtration and distributed mainly in the extracellular fluid [10]. It has been reported that renal function and CCr decrease by approximately 1 mL/min per year of age, while the rate of creatinine production decreases by less than 2 mg/kg/day per decade [35]. Elderly patients frequently exhibit normal serum creatinine concentrations but have less than half the renal function of young adults [36]. Moreover, the ratio of extracellular fluid against intracellular fluid increases in elderly persons, compared with young adults [37]. Therefore, the low clearance and high volume of distribution of amikacin in elderly patients can be attributed to a reduced renal function and increased extracellular fluid, respectively.

It is an important finding from this study that none of the simulated dosing regimens achieved the PK/PD target from EUCAST and the CLSI MIC breakpoint of 16 mg/L. Furthermore, none of the dosing regimens achieved the PK/PD target for susceptible pathogens with an MIC of 16 mg/L. These findings have also been reported by some authors [13,38]. Our results were unable to reveal a single dosing regimen which was suitable for achieving the PK/PD target for both efficacy (Cmax/MIC ≥ 8) against an MIC of 8 mg/L and safety (Cmin < 4 mg/L) for elderly patients. Based on this, we propose optimal dosing regimens using a nomogram based on patient renal function (Table 5). None of the dosing regimens tested achieved the PK/PD target in patients with CCr of < 40 mL/min even when less susceptible pathogens were targeted (MIC of 8 mg/L). The initial dosage for once-daily amikacin needs to be based on CCr; however, there is little data describing dosing regimens suitable for amikacin in patients with CCr of less than 20 mL/min. Gilbert B et al. suggested the dosage and administration interval of amikacin require adjustment for patients who have renal insufficiency [39]. They adjusted only the dosage of amikacin based on CCr to achieve the PK/PD target for safety; however, these regimens did not achieve the PK/PD target for efficacy. In our present study, extended intervals for renal-impaired patients who were administered a dosage equal to patients with normal renal function attained the highest probability of safety without an apparent decrease in efficacy. Surprisingly, there is no study to confirm that dosing interval extension does not decrease the efficacy of amikacin despite the fact that the extended-interval regimen is recommended by multiple studies including our study. However, aminoglycosides including amikacin have a post-antibiotic effect preventing regrowth of bacteria during the period of low antibiotic concentration in serum [40]. Therefore, the extended-interval regimen could be reasonable for elderly patients with renal insufficiency.

Additionally, we assessed the fractional target attainments of the simulated dosing regimens (200–2000 mg every 24–72 h) using the distribution of MIC data of *P. aeruginosa* derived from EUCAST surveillance data. The optimized dosing regimens for P. aeruginosa were the same as our recommended dosing regimens which achieved the PK/PD targets. The latest surveillances on the susceptibility to amikacin of P. aeruginosa isolated from inpatients reported that the MIC90 was 4 mg/L [41]. Therefore, our recommended dosing regimen is enough to cover P. aeruginosa acquired in hospital.

This is the first population PK study to recommend the initial dosing regimen for elderly patients with CCr of 10–90 mL/min. Several limitations should be considered. The data for this study were obtained retrospectively and from a small patient cohort. This may have impacted on our final model not including a covariate to describe an association with the volume of distribution. Furthermore, only two or three blood samples were available for each patient, meaning we were unable to describe a two-compartment model. A limitation of the Pmetrics software is that we were unable to perform a visual predictive check (VPC) with time from dose to evaluate the predictive performance of the model. As such, we constructed the VPC using the dose history of each participant such as in previous studies using the Pmetrics software. However, we provided assurance that our VPC demonstrated the variability and main trends of the observed data. The area under the concentration–time curve (AUC)/MIC ratio is one of the PK/PD targets of amikacin. Although a target value of the AUC/MIC ratio has been reported as an AUC in the range of 0–24 h, that of AUCs in the ranges of 0–48 and 0–72 h has not been reported. Moreover, it has been reported that the AUC/MIC ratio is not associated with clinical response [42], and we also showed the association of Cmax/MIC and clinical outcomes. Therefore, our analysis suggests the dosing regimen based on Cmax/MIC and Cmin. However, since only few studies support the association between AUC/MIC and clinical response, further studies are needed to validate our findings. Finally, French guidelines recommend that the targeted minimum concentration is below 2.5 mg/L [43]. However, we used the minimum concentration of < 4 mg/L to define amikacin-related renal toxicity based on a previous study reporting that the incidence of renal function impairment was significantly higher in elderly patients with a minimum concentration of amikacin of ≥ 4 mg/L [44].

## 4. Materials and Methods

### 4.1. Patients

This study was a single-center retrospective study. All patients admitted to Aichi Medical University Hospital (995 beds) between September 2009 and February 2015 were aged 70 years and above and treated with amikacin for at least 3 days. The blood concentrations of amikacin from patients were obtained as part of routine clinical practice of therapeutic drug monitoring (TDM) in our hospital. The patients with intermittent and continuous renal replacement therapy at the onset of amikacin therapy or who did not measure both minimum and maximum concentrations were excluded. Ethical approval was obtained from the ethics committee of the Aichi Medical University (No. 14-053).

### 4.2. Data Collection

All clinical data were extracted from the electronic health records through structured chart review. Demographic data at least 3 days before amikacin treatment started were collected, including gender, age, total weight, lean body weight (LBW), body mass index (BMI), ideal body weight (IBW), albumin, serum creatinine, CCr estimated according to the Cockcroft–Gault equation [45] since the Cockcroft–Gault equation is the best predictive equation of creatinine clearance for elderly patients [46], aspartate aminotransferase (AST), alanine aminotransferase (ALT), blood urea nitrogen (BUN), and total bilirubin. IBW was calculated as follows: IBW = 22 × (height in m)^2^ (kg). LBW, in kilograms, was calculated by the method of Janmahasatian and colleagues: LBW (males) = (9270 × body weight)/[6680 + (216 × BMI)]; LBW (females) = (9270 × body weight)/[8780 + (244 × BMI)] (kg) [47]. Likewise, TDM data were collected, including the period of its treatment, the dosage, infusion time, amikacin blood concentration, and infusion and sampling times. All blood samples were obtained at the following times: (i) within 30 min before amikacin administration (Cmin) and (ii) approximately 1 h after amikacin administration started (Cmax). Moreover, we evaluated the 30-day mortality and microbiological eradication.

Amikacin concentrations were measured by a fluorescence polarization immunoassay with the amikacin assay kit (Roche Diagnostics K., Tokyo, Japan). The limit of detection was 0.8 mg/L, and the coefficients of intra- and inter-assay variation were within 6% across the whole calibration range (0.8 to 40.0 mg/L).

### 4.3. Population Pharmacokinetic Modeling

As previously reported [38], one- and two-compartment models were analyzed with the nonparametric adaptive grid (NPAG) algorithm within the Pmetrics software package (version 1.5.2) for R (Laboratory of Applied Pharmacokinetics, University of Southern California, Los Angeles, CA, USA) [48,49]. We modeled elimination from the central compartment and the intercompartmental distribution into the peripheral compartment.

### 4.4. Population Pharmacokinetic Covariate Building

The influence of the following covariates at initiation of treatment on amikacin pharmacokinetic parameters was evaluated: demographic variables (gender, age, total weight, LBW, BMI, and IBW) and biochemical markers (albumin, serum creatinine, eGFR, CCr, AST, ALT, and BUN). A covariate that improved the log-likelihood (−2×LL) value by more than 3.84 and improved the goodness-of-fit plots was included.

### 4.5. Model Evaluation

Evaluation of the final model was conducted using statistical and graphical methods. Inclusion of primary model parameters was based on a decrease in log-likelihood values by more than 3.84 and a decrease in the Akaike information criterion (AIC). Goodness of fit was evaluated based on linear regression with an improvement in the observed-predicted plot and coefficients of determination. Predictive performance was assumed by the mean prediction error (bias) and the mean bias-adjusted squared prediction error (imprecision) for the population and individual prediction models. Moreover, the calculation of the weighted residual errors against observed concentrations and time after dose was used to evaluate the model. A bootstrapping method by simulation of 1000 subjects was used to assess the stability of the final model and the precision of parameter estimates [50], with an a priori target of > 90% for the proportion of observations found within the 5th and 95th percentiles deemed acceptable. A visual predictive check (VPC) was performed using the bootstrapping method to evaluate the predictive performance of the model.

### 4.6. Assessment of Amikacin Dosing Regimens by Monte Carlo Simulation

Monte Carlo simulations were employed using Pmetrics to estimate the distribution of amikacin PK parameters for 1000 subjects in the final model with covariates. To assess amikacin maximum concentrations, we simulated the concentration obtained 1 h after the start of 30 min infusion for several dosing regimens (200–2000 mg). We compared the PTA of a Cmax/MIC ≥ 8 [8,51] for MICs of 4, 8, and 16 mg/L in patients with CCr of 10–90 mL/min. For an assessment of amikacin minimum concentrations, we simulated the concentration at 23.5 (day 1), 47.5 (day 2), and 71.5 (day 3) hours after the start of amikacin infusion, which considered the regimens of once per 24, 48, and 72 h (q24h, q48h, and q72h) for several dosing regimens (200–2000 mg). We determined the probability of target attainment (PTA) for a Cmin ≤ 4 mg/L [13] in patients with CCr of 10–90 mL/min. This timing for amikacin sampling is commonly used for studying aminoglycoside efficacy and safety [9,52].

MIC data for Pseudomonas aeruginosa obtained from the EUCAST database [53] were used to calculate fractional target attainment. Fractional target attainment is a clinically relevant descriptor of the likely success of treatment by comparing the PTA against an MIC distribution. In particular, we focused on dosing regimens to achieve the PK/PD target for both efficacy (Cmax/MIC ≥ 8) and safety (Cmin < 4 mg/L).

### 4.7. Statistical Analysis

Statistical significance of the difference between two groups was assessed by a chi-squired test for categorical data. Data were analyzed with JMP version 10.0 (SAS, Tokyo, Japan). A *p* value of <0.05 was required to reach statistical significance.

## 5. Conclusions

Our study provides rational evidence relevant to initial dosing regimens of amikacin in elderly patients. We recommend dosing regimens for amikacin following a nomogram based on CCr for an MIC of ≤ 8 mg/L for elderly patients with CCr of 40–90 mL/min. We are unable to recommend dosing for elderly patients with CCr of < 40 mL/min. Furthermore, we do not recommend amikacin monotherapy for a pathogen with an MIC of 16 mg/L.

## Figures and Tables

**Table 1:** Clinical characteristics and distribution of amikacin of hospitalized patients.

Parameter	Mean ± SD	Median [Range]
Male/female	6/9	-
Age (year)	80.6 ± 7.3	80.0 [71–95]
Weight (kg)	44.8 ± 8.9	42.6 [32.5–67.3]
Lean body weight (kg)	35.5 ± 8.1	34.6 [24.6–51.1]
BMI (kg/m^2^)	19.1 ± 3.0	19.7 [14.9–25.6]
Ideal body weight (kg)	51.9 ± 7.4	51.5 [40.1–68.1]
Albumin (g/dL)	2.5 ± 0.4	2.4 [1.7–3.2]
Serum creatinine (mg/dL)	0.84 ± 0.77	0.59 [0.32–3.39]
CCr * (mL/min)	52.9 ± 22.8	52.1 [10.9–94.9]
AST (U/L)	46 ± 58	31 [6–243]
ALT (U/L)	32 ± 23	26 [7–91]
BUN (mg/dL)	24.8 ± 19.7	16.4 [6.8–71.6]
Total bilirubin (mg/dL)	1.10 ± 0.88	0.65 [0.38–3.16]
Duration of amikacin therapy (days)	9 ± 6	7 [3–20]
Amikacin dosage (mg/day)	440 ± 226	400 [200–1000]
Amikacin dosage (mg/kg/day)	9.6 ± 3.6	9.4 [4.3–17.7]
Infusion time (h)	0.67 ± 0.41	0.5 [0.5–1.0]

SD, standard deviation; CCr, creatinine clearance; *, CCr calculated according to the Cockcroft–Gault equation.

**Table 2:** Final estimates of population pharmacokinetic parameters for amikacin.

	Mean	SD	Median	SE	CV%	Var	Shrink%
CL (L/h)	2.25	0.78	2.19	0.24	34.6	2.19	4.20
V (L)	18.0	3.4	17.1	1.0	18.9	17.1	17.22

SD, standard deviation; SE, standard error; CV, coefficient of variation; Var, variance.

**Table 3:** Monte Carlo simulations and probability of target attainment (PTA) of toxicity (Cmin < 4 mg/L) and efficacy (Cmax/MIC ≥ 8) against an MIC of 4 mg/L for various amikacin dosing regimens (200–2000 mg) for patients with CCr of 10–90 mL/min.

(A). q24h
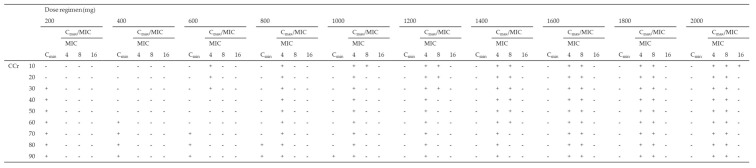
(B). q48h
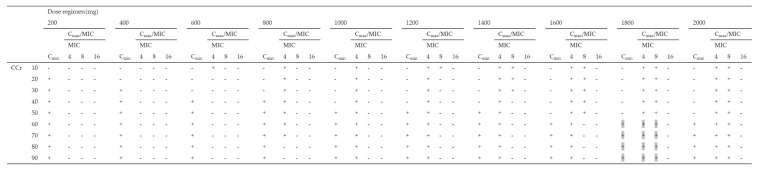
(C). q72h
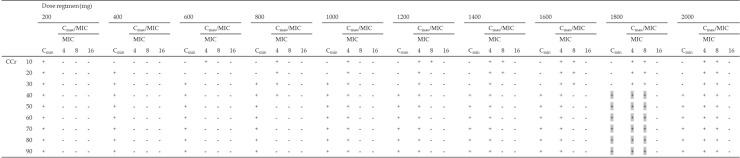

**Table 4:** Fractional target achievement for amikacin dosing regimens (200–2000 mg) for patients with CCr of 10–90 mL/min for susceptible MIC distributions for *P. aeruginosa*.

**(A). q24h**											
		**Dose regimen (mg)**									
		**200**	**400**	**600**	**800**	**1000**	**1200**	**1400**	**1600**	**1800**	**2000**
CCr	10	32.6	68.0	77.2	88.9	93.5	93.9	95.7	98.1	98.7	99.3
	20	16.9	49.9	76.3	81.1	90.9	93.6	93.8	95.2	96.9	98.6
	30	10.0	41.9	75.7	77.1	85.4	93.3	93.6	93.9	95.3	96.7
	40	8.5	40.1	70.4	76.4	82.2	90.8	93.6	93.6	94.3	95.8
	50	8.3	39.9	65.1	76.4	79.8	88.3	93.3	93.6	93.9	94.9
	60	8.3	39.9	62.3	76.3	77.9	86.9	92.4	93.6	93.7	94.4
	70	8.3	39.8	59.8	76.2	77.0	85.8	91.6	93.6	93.7	94.1
	80	8.2	39.3	58.0	75.5	76.6	84.9	91.1	93.3	93.6	93.9
	90	8.0	38.2	55.9	74.5	76.5	83.9	90.7	92.7	93.6	93.8
**(B). q48h**											
		**Dose regimen (mg)**									
		**200**	**400**	**600**	**800**	**1000**	**1200**	**1400**	**1600**	**1800**	**2000**
CCr	10	18.2	51.3	76.4	81.5	91.5	93.6	93.9	95.4	97.0	98.6
	20	8.9	40.6	72.1	76.4	83.5	91.9	93.6	93.7	94.6	96.1
	30	8.4	40.0	64.8	76.3	79.3	88.7	92.6	93.6	94.0	94.9
	40	8.4	39.8	62.2	76.3	77.4	87.4	91.5	93.5	93.9	94.5
	50	8.0	38.3	60.4	74.6	76.8	86.5	91.2	92.7	93.8	94.3
	60	7.7	36.7	58.8	73.4	76.6	85.6	91.0	91.9	93.7	94.1
	70	7.6	36.1	57.0	72.6	76.5	84.7	90.7	91.6	93.6	94.0
	80	7.6	36.0	55.7	72.5	76.5	83.6	90.5	91.5	93.6	93.9
	90	7.6	36.0	54.1	72.5	76.4	82.7	90.1	91.5	93.3	93.8
**(C). q72h**											
		**Dose regimen (mg)**									
		**200**	**400**	**600**	**800**	**1000**	**1200**	**1400**	**1600**	**1800**	**2000**
CCr	10	11.1	43.1	75.8	77.2	87.1	93.3	93.6	94.1	95.6	97.1
	20	8.7	40.3	66.7	76.3	80.2	89.3	92.7	93.7	94.0	95.0
	30	8.5	39.7	64.0	75.9	77.9	88.1	91.5	93.4	93.9	94.4
	40	7.9	37.2	61.9	73.2	77.1	86.9	91.4	91.6	93.8	94.2
	50	7.8	36.4	60.1	72.4	76.8	86.2	91.2	91.6	93.7	94.1
	60	7.7	36.2	58.8	72.3	76.6	85.5	90.9	91.6	93.6	94.0
	70	7.6	36.2	57.1	72.2	76.5	84.5	90.7	91.6	93.4	93.9
	80	7.6	36.2	55.7	72.2	76.4	83.8	90.5	91.6	93.2	93.8
	90	7.6	36.2	53.8	72.2	76.4	82.9	90.1	91.6	93.0	93.8

q24h, q48h, and q72h, administered every 24, 48, and 72 h, respectively. Gray shading, dosing regimen to achieve the priori PTAs of both Cmax/MIC of ≥ 8 and Cmin of < 4 mg/L.

**Table 5:** Recommended amikacin dosing regimens for patients with CCr of 10–90 mL/min.

Renal Function	CCr	Recommended Dose Regimen
Kidney failure	10	NA
	20	NA
Severe impairment	30	NA
	40	1800 mg q72h
Moderate impairment	50	1800 mg q72h
	60	1800 mg q48h
Mild impairment	70	1800 mg q48h
	80	1800 mg q48h
Normal	90	1800 mg q48h

q24h NA, no recommended dosing regimen. q24h, q48h, and q72h, administered every 24, 48, and 72 h, respectively.

## Data Availability

The datasets analyzed during this study are available and can be obtained, at request, on reasonable enquiry.
